# A New High-Pressure High-Temperature Phase of Silver Antimonate AgSbO_3_ with Strong Ag–O Hybridization

**DOI:** 10.1021/acs.inorgchem.4c03021

**Published:** 2024-11-11

**Authors:** Mohamed Oudah, Minu Kim, Robert Dinnebier, Graham McNally, Kateryna Foyevtsova, Doug Bonn, Hidenori Takagi

**Affiliations:** † Max Planck Institute for Solid State Research, Heisenbergstrasse 1, 70569 Stuttgart, Germany; ‡ Stewart Blusson Quantum Matter Institute, University of British Columbia, Vancouver, BC V6T 1Z4, Canada

## Abstract

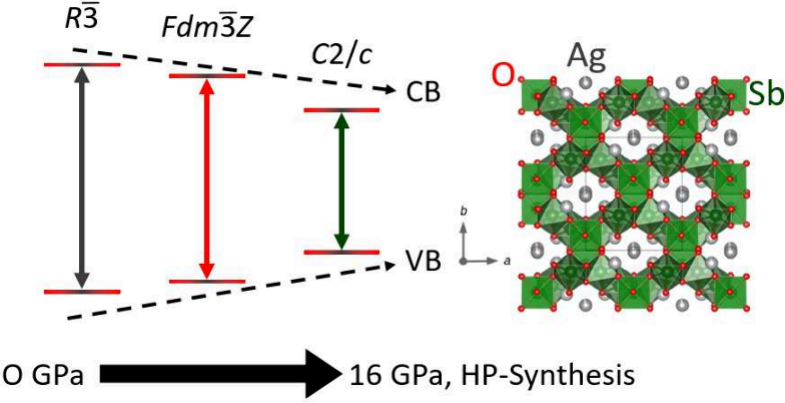

We report on a new polymorph of silver antimonate AgSbO_3_ discovered with the use of high-pressure high-temperature synthesis at 16 GPa and 1380 °C. The crystal structure is determined from X-ray powder diffraction, and we find this new high-pressure phase crystallizes in monoclinic space group *C*2/*c* with the following values: *a* = 8.4570(3) Å, *b* = 9.8752(3) Å, *c* = 8.9291(3) Å, β = 91.1750(12)°, and *V* = 745.56(4) Å^3^. We synthesized the high-pressure (16 GPa) AgSbO_3_ phase from the ilmenite phase as a precursor. This high-pressure monoclinic AgSbO_3_ consists of a three-dimensional network of corner- and edge-sharing SbO_6_ octahedra with channels along the *c*-direction containing Ag atoms. We also synthesize AgSbO_3_ in the defect pyrochlore phase at 4 GPa from the same ilmenite precursor and compare the Raman spectra and the cation–anion bonding of all three AgSbO_3_ phases. The absence of a cubic perovskite form of AgSbO_3_ even at pressures of ≤16 GPa is likely due to the covalency of the Sb–O bonds and the moderate electronegativity of Ag^+^. Hybridization of Ag d and O p orbitals results in a variation of Ag–O distances that correlates with the band gap, which is in qualitative agreement with the density of states around the Fermi level from our density functional calculations. We compare AgSbO_3_ with other ABX_3_ compounds to elucidate the dependence of the structure on the constituent atoms.

## Abstract 2

High-pressure (HP) AgSbO_3_ syntheiszed at 16 GPa and 1380 °C exhibits a monolinic *C*2/*c* space group. Compared with the ilmenite (*R*3̅) phase and defect pyrochlore (*Fd*3̅*mZ*), we find a smaller band gap in the monoclinic HP-AgSbO_3_ phase (*C*2/*c*). In all three phases, our density functional theory calculations reveal that the top of the valence band and the bottom of the conduction band are dominated by contributions from strong Ag−O hybridized states.

## Introduction

Perovskite-related structures have unique electronic and magnetic properties, making them of great interest for technological applications. Typically, for a composition of ABX_3_, where A and B are cations and X is an anion, the stability of the perovskite structure is evaluated on the basis of the relative sizes of the ions,^1^ and many stable compositions for X = O^2–^ or F^–^ are known.^2,3^ While many ABO_3_ oxides crystallize in the cubic perovskite structure with corner-sharing BO_6_ octahedra and a B–O–B linkage of 180°, the A^+^Sb^5+^O_3_ compounds do not form structures having Sb–O–Sb groups with 180° linkages due to the covalency of the Sb–O bond.^4^ All known A^+^Sb^5+^O_3_ compounds crystallize in structures in addition to the cubic perovskite, including ilmenite and defect pyrochlore, when synthesized under ambient pressure. We note that the cubic perovskite structure can be stabilized for  for *x* ≥ 0.65 under high-pressure conditions,^5^ where the average oxidation state of Sb is less than 5+ with the addition of Ba^2+^. However, here we limit the discussion to materials with only one element on the A site and one element on the B site, specifically antimonates for which B = Sb^5+^, and the stability of the cubic perovskite structure.

The cubic perovskite structure can be stabilized under high-pressure conditions for many  compounds for which B = Ge and Sn, where the size of the Ge^4+^ or Sn^4+^ ion is comparable to that of Sb^5+^.^6^ In addition, under high-pressure high-temperature conditions, the orthorhombic perovskite structure was reported for the antimonate NaSbO_3_ at 10.5 GPa and 1150 °C.^7^ However, even under higher pressures, we are unable to stabilize a perovskite structure for AgSbO_3_ but instead found a new monoclinic phase was stabilized at 16 GPa and 1380 °C. A number of AgSbO_3_ phases are reported in the literature, including an ilmenite phase synthesized through ion exchange under ambient conditions^8^ and a defect pyrochlore phase synthesized at 0.3–6.5 GPa.^9^

**Figure 1:**
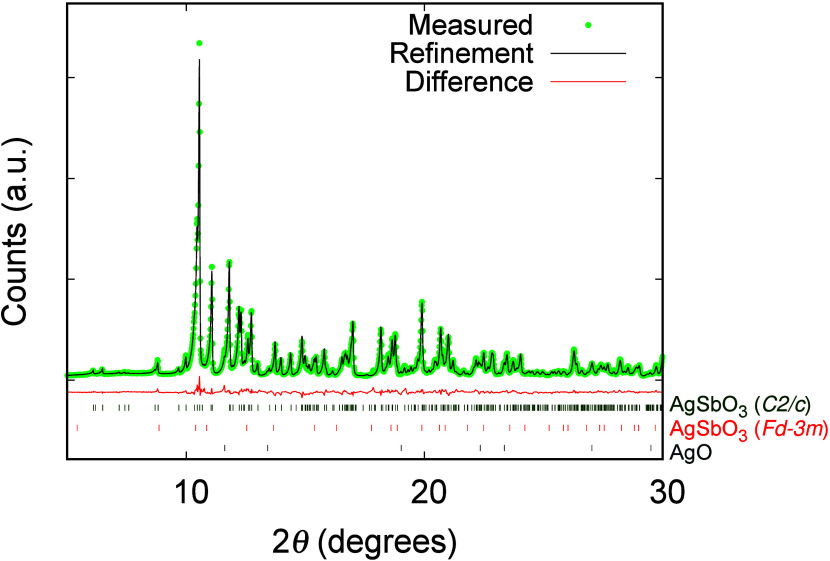
Scattered X-ray diffraction intensities of high-pressure monoclinic AgSbO_3_ (*C*2/*c* symmetry) [with AgO and AgSbO_3_ as impurity phases with 0.9 and 0.5 wt %, respectively] at 298 K under ambient conditions as a function of 2θ. The observed pattern (blue circles) measured in Debye–Scherrer geometry, the best combined Rietveld fit profiles (red line), and the difference curves between the observed and calculated profiles (gray line) are shown. The peak positions of the three phases are presented as blue lines below. The square root of the intensity is shown to better visualize the smaller reflections.

Non-perovskite ABO_3_ oxides can have advantageous properties, such as high ionic conductivity due to the low-symmetry structures that allow hopping between ionic sites,^10^ which is absent in perovskite-type ABO_3_. This monoclinic AgSbO_3_ phase crystallizes in space group *C*2/*c*, and its X-ray diffraction pattern and Rietveld refinement are shown in Figure 1. We compare the crystal structure, optical conductivity, and Raman spectra of this high-pressure monolinic phase with those of the defect pyrochlore and ilmenite phases of AgSbO_3_.

In the monoclinic AgSbO_3_ phase, we find a combination of corner- and edge-sharing SbO_6_ octahedra forming a three-dimensional network with channels running along the *c*-direction, while the Ag atoms occupy positions inside these channels. We compare Ag–O and Sb–O coordination in all of these phases and discuss the absence of a cubic perovskite phase even under high-pressure high-temperature conditions in relation to the covalency of the Sb–O bond and the moderate electronegativity of Ag^+^. We contrast our findings with those of other perovskite oxides with a similar ionic size at the B site and those of the cubic perovskite NaSbO_3_ phase stabilized at high pressure.

## Results and Discussion

**Figure 2:**
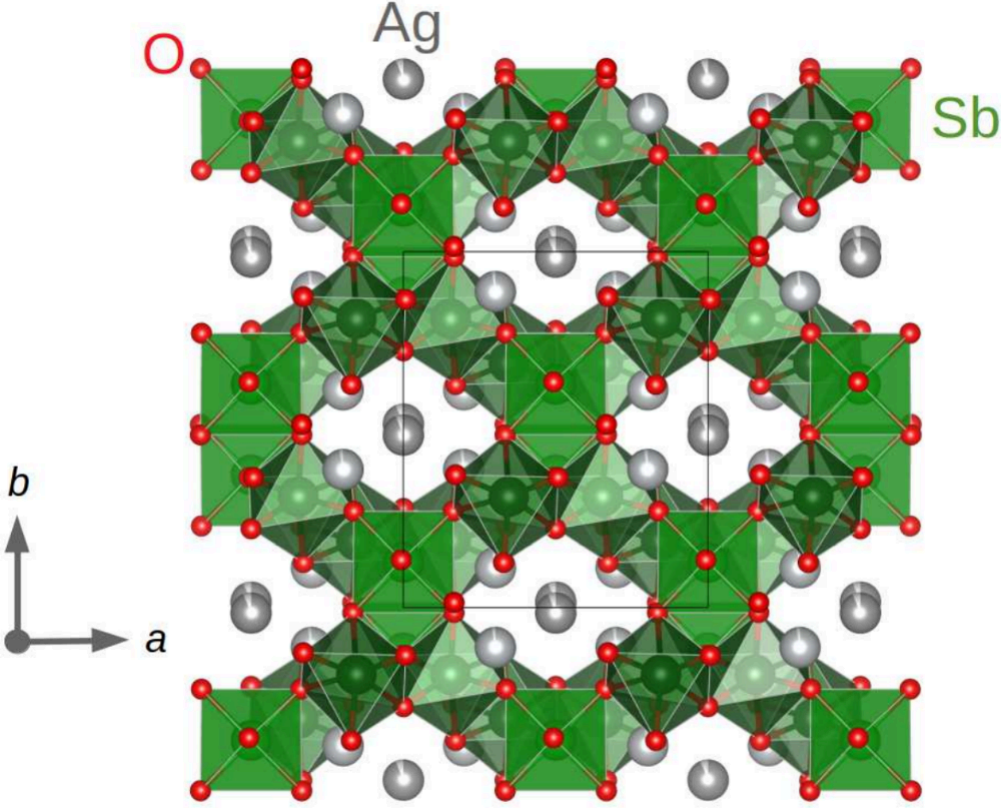
Projection of the crystal structure of monoclinic AgSbO_3_ (*C*2/*c* symmetry) in the *a–b* plane.

**Figure 3:**
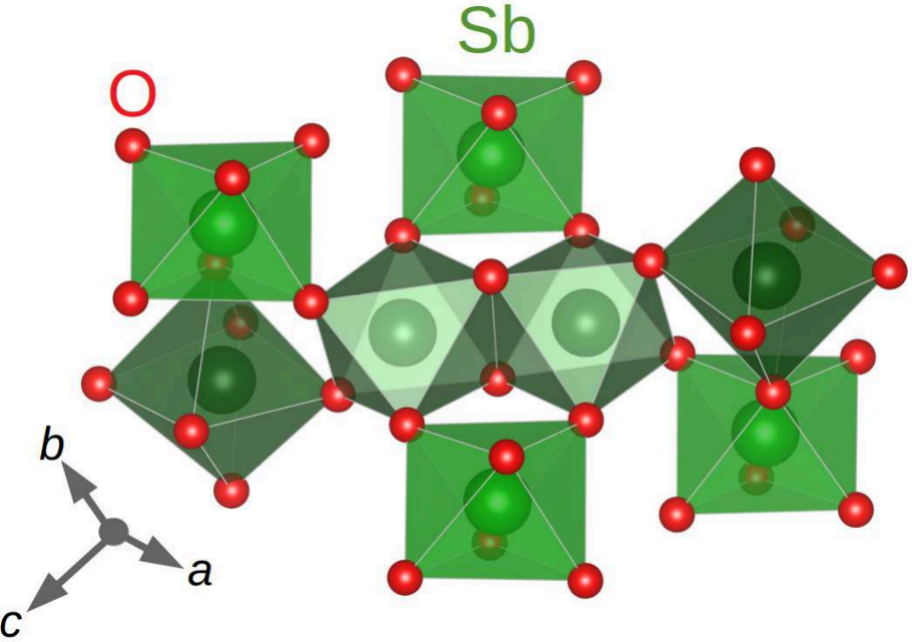
Connectivity of SbO_6_ octahedra forming the framework in the crystal structure of monoclinic AgSbO_3_.

The crystal structure of monoclinic AgSbO_3_ (Figure 2) is fully ordered and can best be described as follows. Corner-sharing Sb_2_O_10_ double octahedra form a subunit with an inversion center inside (Figure 3). The two upper and lower vertices of the double octahedra are connected to the edge of neighboring SbO_6_ octahedra, which are thus turned by 90°. In addition, the four outer corners of the double octahedra are corner shared with two corner-sharing pairs of additional SbO_6_ octahedra that are connected to other Sb_2_O_10_ double octahedra thus forming a rigid three-dimensional porous network with interconnected pseudohexagonal channels along the *c*-direction. The results of the Rietveld refinement are listed in Table 1, and the atomic positions are listed in Table S1.

**Table 1:** Crystallographic and Rietveld Refinement Data of High-Pressure Monoclinic AgSbO_3_^a^

cell mass (g mol^–1^)	3366(11)
crystal system	monoclinic
space group	*C*2/*c*
wavelength (Å)	0.55941
*a* (Å)	8.4570(3)
*b* (Å)	9.8752(3)
*c* (Å)	8.9291(3)
α (deg)	90
β (deg)	91.1750(12)
γ (deg)	90
*V* (Å^3^)	745.56(4)
*T* (K)	298
*Z*	8
*D*_calc_ (g cm^–3^)	7.420
*R*_wp_ (%)	6.02
*R*_p_ (%)	4.37
*R*_Bragg_ (%)	1.73
measured starting angle 2θ (deg)	2
measured final angle 2θ (deg)	112
starting angle 2θ used (deg)	4
final angle 2θ used (deg)	62
step width (deg 2θ)	0.015
time (h)	12

a *R*_wp_, *R*_p_, and *R*_Bragg_ are as defined in TOPAS version 7.^11^

To gain insight into this crystal structure, we examined the ABO_3_ oxides, where the B cation prefers octahedral coordination. The most stable structure depends on the ionic or covalent nature of the bonding. In the case of covalent bonding, we must consider the σ-bonding and the π-bonding between the B and O ions. Ionic forces between the atoms tend to favor corner-sharing cubic perovskite structure and have been suggested to explain the stability of the perovskite phases. AGeO_3_ and ASnO_3_ compounds crystallize in the cubic perovskite phase, some stable only under high pressure.^12,13^ The Shannon radii of octahedrally coordinated Ge^4+^ and Sn^4+^ on the B site are 0.67 and 0.83 Å, respectively, while Sb^5+^ has a radius of 0.74 Å.^6^ With A^+^ and A^2+^ ions with similar radii available among group I and group II elements, we must consider effects beyond the cation size to explain the lack of stability of a cubic perovskite structure of A^+^SbO_3_.

The electronegativity of Sb^5+^ relative to that of Ge^4+^ and Sn^4+^ ions can explain the edge sharing found in ASbO_3_. We find that edge sharing of octahedra is preferred in all ASbO_3_ compounds synthesized under ambient conditions. In monoclinic AgSbO_3_, we find the Sb–O–Sb group with linkages of 94.1° and 119.8°, and this deviation from a linear Sb–O–Sb group is consistent with strong covalent Sb–O bonding. Considering covalent bonding, linear Sb–O–Sb bonding would require the same O 2p orbital to participate in σ-bonding to two Sb atoms. However, the covalent bonding would be much stronger if different anionic orbitals were to participate in the bonding with each of the cations. This is consistent with the observation of edge-sharing octahedra with a Sb–O–Sb angle of 90° found in many ASbO_3_ compounds.

**Figure 4:**
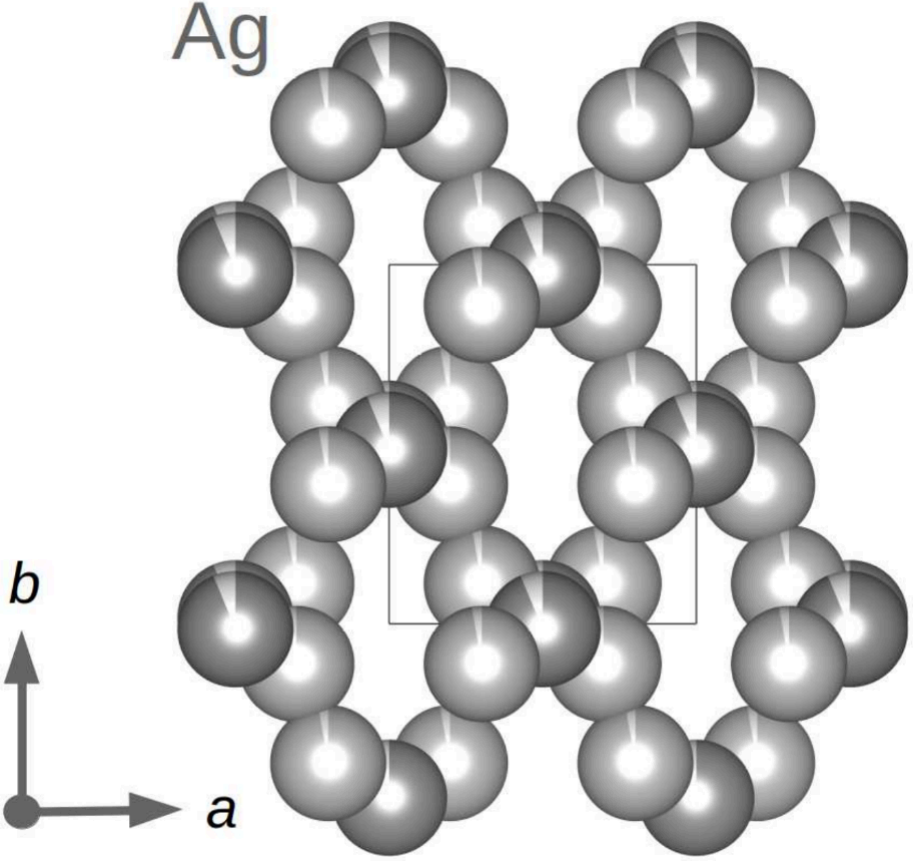
Three-dimensional silver network in the crystal structure of monoclinic AgSbO_3_ (*C*2/*c* symmetry).

Via application of a pressure of 10.5 GPa, NaSbO_3_ can be stabilized as an orthorhombically distorted perovskite, where the Na^+^ and [SbO_3_]^−^ ionic interactions allow for the stabilization of the perovskite phase.^7^ In monoclinic AgSbO_3_, we go to an even higher pressure of 16 GPa and might expect a similar Ag^+^ and [SbO_3_]^−^ interaction to facilitate the stability of the perovskite structure. However, the instability of the perovskite phase even at these pressures is likely due the weaker ionic interaction of [SbO_3_]^−^ with Ag^+^ than with Na^+^. As discussed below, the more covalent nature of Ag^+^ is evidenced by the color of the samples relative the Ag^+^–O distance as discussed below, and we find a variation in the oxygen coordination of Ag^+^. The Ag atoms are located in the channels and are interconnected in a three-dimensional fashion with Ag–Ag distances between 2.866 and 3.085 Å (Figure 4); such a network is expected to facilitate Ag^+^ hopping, resulting in significant ionic conductivity.

We examine the Ag–O distances in all three AgSbO_3_ phases and find a shortest Ag–O distance of 2.220 Å in monoclinic AgSbO_3_. The shortest Ag–O distances in the ilmenite and defect pyrochlore phases are 2.414 and 2.555 Å, respectively. These distances are consistent with the correlation between the Ag–O distance and color in various Ag oxides discussed previously.^4^ The O coordination around the Ag atoms, which can also effect the band gap due to the change in orbital overlap, varies between the ilmenite and defect pyrochlore phases and is highly asymmetric in monoclinic AgSbO_3_. We find a variation in the Ag^+^–O bonding with the Ag1 site having one O atom with a distance of 2.220 Å and two O atoms with a distance of 2.514 Å in a triangular planar configuration. For the Ag2 site, we find O atoms at distances of 2.393, 2.414, and 2.498 Å in a deformed trigonal pyramidal configuration. This uneven bonding can originate from the coordinated covalency of oxygen atoms that results in deformation of the 4d^10^ orbital of the Ag^+^ ion due to 4d–5s hybridization.

**Figure 5:**
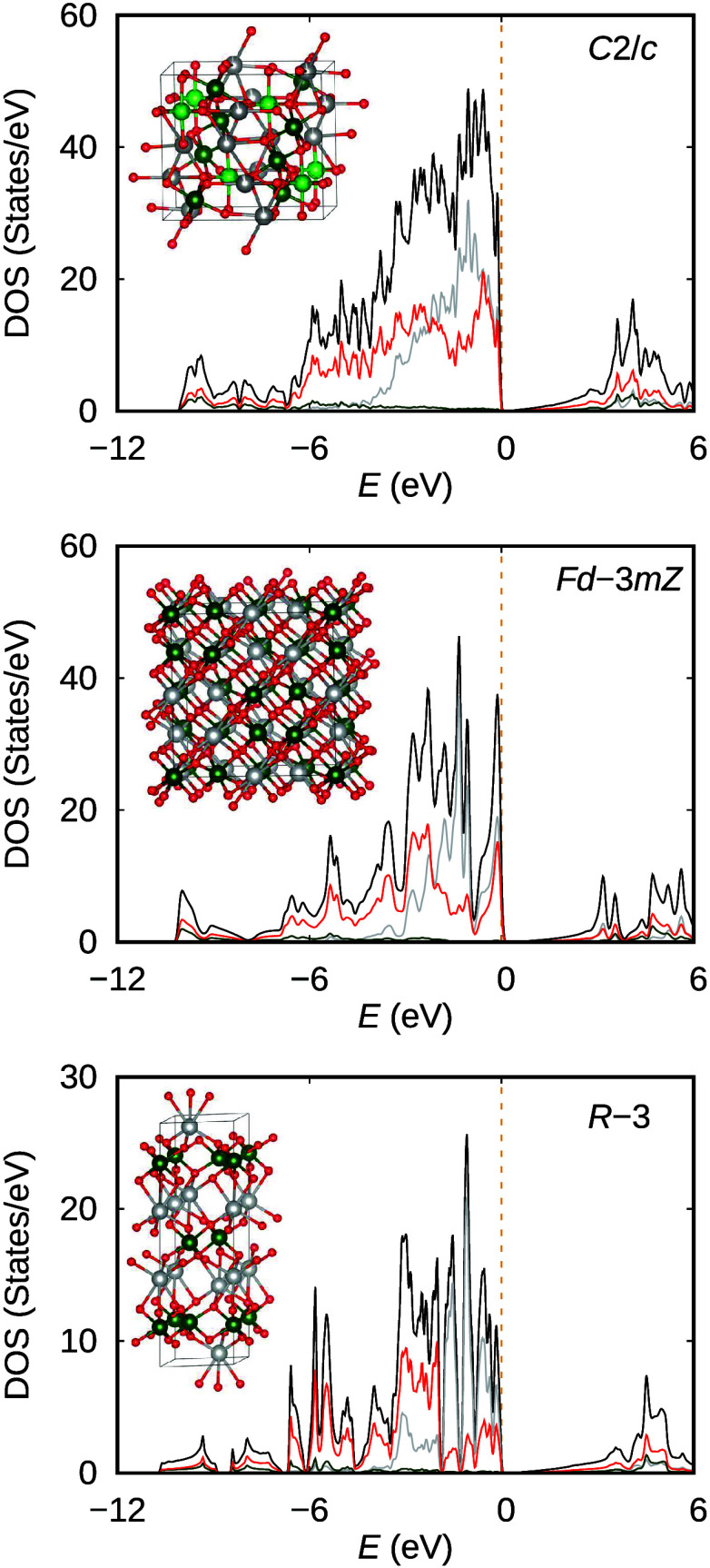
Density of states of all three AgSbO_3_ phases: ilmenite (synthesized at ambient pressure, *R*3̅ symmetry), defect pyrochlore (synthesized at 4 GPa, *Fd*3̅*mZ* symmetry), and monoclinic phase (synthesized at 16 GPa, *C*2/*c* symmetry). The total density of states (black) and partial contributions from Ag (gray), Sb (green), and O (red) are highlighted.

From density functional calculations, we look at the density of states (DOS), shown in Figure 5, for all three AgSbO_3_ phases. From the partial contribution of Ag, Sb, and O, we indeed find that Ag and O contribute heavily below the *E*_f_. Also, we find the peaks for both Ag and O appear at similar energies, supporting the scenario for suspected strong hybridization based on the short Ag–O distance. This is especially evident for the ilmenite and defect pyrochlore phases with single Ag and O sites. Upon examination of the band structure in Figure S3, the density of bands below the Fermi level increases in the *C*2/*c* symmetry due to the increased number of unique sites, with two Ag sites, two Sb sites, and five O sites. We examine the partial DOS for the monoclinic AgSbO_3_ phase in Figure S4 considering the shortest distance between cations and oxygen anions. We find the shortest distance between the nearest O atoms and Ag1, Ag2, Sb1, and Sb2 corresponds to peaks in the partial DOS. The O4 site seems to be strongly bonded to the Ag1 site and has a short distance of 2.221 Å. However, this distance does not necessarily reflect the strongest bonding between Ag1 and O4, because O4 is strongly bonded to the Sb2 atom as part of the surrounding O atoms in an octahedral arrangement. This results in deformation of the core 4d^10^ orbital from a sphere to an ellipsoid, allowing for shorter bonds along the shorter directions of the ellipsoid. Such a variation in Ag–O bonds was previously reported for Ag^+^ salts and discussed for the cubic *Im*3̅ phase of AgSbO_3_.^4^ The color of AgSbO_3_ samples reflects the band gap, *E*_g_, where an *E*_g_ of <1.7 eV results in black compounds and an *E*_g_ of >3.0 eV results in white compounds.^4^

**Figure 6:**
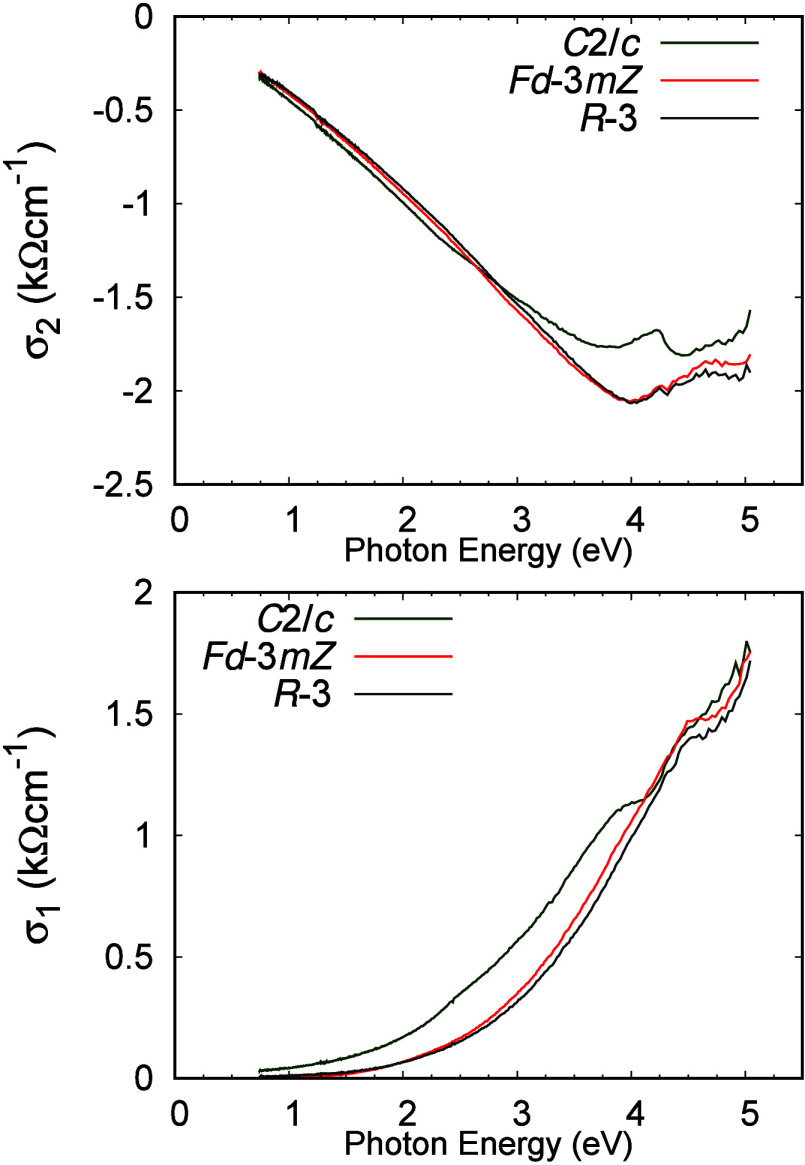
Real (σ_1_) and imaginary (σ_2_) parts of the electrical conductivity of AgSbO_3_, ilmenite (synthesized at ambient pressure, *R*3̅ symmetry), defect pyrochlore (synthesized at 4 GPa, *Fd*3̅*mZ* symmetry), and the monoclinic phase (synthesized at 16 GPa, *C*2/*c* symmetry).

The change in band gap with Ag–O bond length can be understood if nonbonding core orbitals, 4d or hybridized 4d–5s of the Ag^+^, contribute to the top of the valence band and 5s orbitals contribute to the bottom of the conduction band. Hybridization will increase the 4d levels relative to the 5s levels, thus decreasing the gap between the 4d-like bands and the 5s-like bands. Reduction of the Ag–O bond length requires greater 4d–5s hybridization; therefore, we find a decrease in the energy gap with a decrease in the Ag–O bond length. This is consistent with our findings from ellipsometry measurements, shown in Figure 6, for determining the optical conductivities of all three AgSbO_3_ phases. The ilmenite phase has the largest band gap of 2.50 eV, which is slightly reduced to 2.30 eV in the defect pyrochlore phase and much reduced to 1.90 eV in the monoclinic AgSbO_3_ phase. This supports the strong 4d–5s hybridization of the Ag^+^ suspected from the Ag–O bonding based on the crystal structure. The band structure in Figure S3 shows a band above the Fermi level that is separated by only 100 meV from the valence band. However, this band may be optically inactive, or its position appears closer to the Fermi level due to the functionals used, which results in an underestimation of the optical band gap. Considering the number of distinct sites in the monoclinic *C*2/*c* phase, this band likely comes from a cluster of atoms that extends across the unit cell. We utilize a molecular orbital approach to examine this possibility as demonstrated in Figure S5 and find the band above the Fermi level is dominated by Ag and O contributions. This confirms the Ag–O hybridization suspected on the basis of atomic distances and the change in the observed optical band gap.

**Figure 7:**
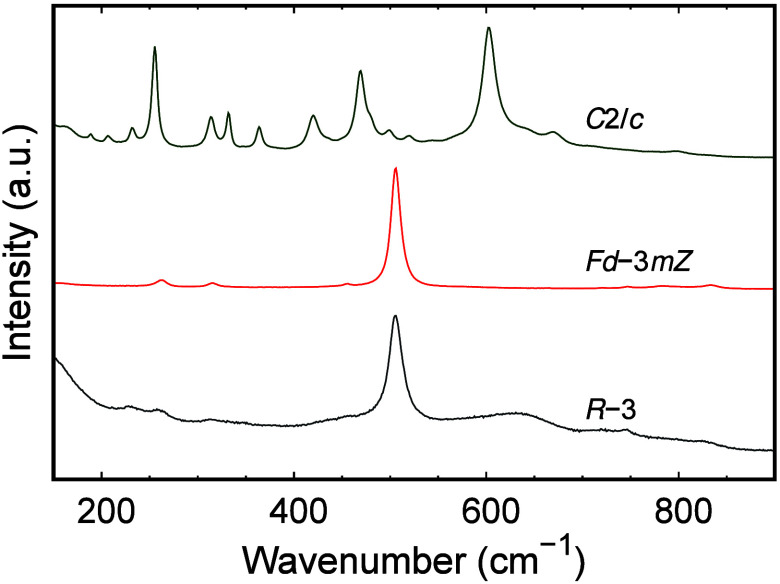
Raman spectra of different phases of AgSbO_3_, ilmenite (synthesized at ambient pressure, *R*3̅ symmetry), defect pyrochlore (synthesized at 4 GPa, *Fd*3̅*mZ* symmetry), and the monoclinic phase (synthesized at 16 GPa, *C*2/*c* symmetry).

We examine the Raman spectra of all three phases in Figure 7, measured without any polarizers in the range of 100–800 cm^–1^. We find similar spectra for the ilmenite and defect pyrochlore phases with a strong peak at ∼500 cm^–1^. For the monoclinic AgSbO_3_ phase, we find richer spectra with many peaks, where the strongest peaks are at 250 and 600 cm^–1^. The strongly bonded Sb–O network is likely to affect the Raman spectra in all three phases. We find only one Sb site and one O site in the ilmenite and defect pyrochlore phases, whereas we have two Sb sites and five O sites in the monoclinic AgSbO_3_ phase.

The large number of distinct Sb and O sites and the low symmetry in the monoclinic AgSbO_3_ phase (*C*2/*c*) are consistent with the large number of optical modes observed in the Raman spectra. With 30 independent atoms in the primitive unit cell, we have 3*n* = 90 phonon modes in monoclinic AgSbO_3_, with three accoustic modes and 87 optical phonon modes, where 45 are Raman active optical phonons. Denoted by the irreducible representations, for the monoclinic *C*2/*c* phase of AgSbO_3_ with the *C*_2*h*_(2/*m*) point group we have 21 A_g_ and 24 B_g_ modes.^14^ To characterize the Raman spectra in more detail and identify the different optical modes, calculations and further polarization-dependent Raman experiments will be needed. The low symmetry of the crystal structure and the large change in the DOS below the Fermi level may result in the good performance of this material for thermoelectric applications.

## Conclusion

We find a new polymorph of AgSbO_3_ stabilized using a high-pressure, high-temperature synthesis technique at 16 GPa and 1380 °C from an ilmenite phase as the starting material. The crystal structure is determined from XRPD to be in the monoclinic *C*2/*c* space group with two Sb sites and five oxygen sites. This monoclinic AgSbO_3_ consists of a three-dimensional network of corner- and edge-sharing SbO_6_ octahedra with channels along the *c*-direction containing two crystallographically distinct Ag cations. Upon comparison with AgSbO_3_ in the defect pyrochlore phase synthesized at 4 GPa and the ilmenite phase, we find edge-sharing SbO_6_ octahedra in all three phases.

The Ag–O bonding varies in all three phases, which is expected to change the degree of 4d–5s hybridization and the resulting band gap. Indeed, we find a reduction in the optical band gap in our ellipsometry measurement with a shorter Ag–O distance, with the smallest band gap of 1.90 eV in monoclinic AgSbO_3_. The Raman spectra were recorded for all three AgSbO_3_ phases, and we find a rich spectrum for monoclinic AgSbO_3_ with many peaks, which is consistent with increased number of Sb sites and O sites compared with the ilmenite and defect pyrochlore phase showing a single dominant peak. The absence of cubic perovskites in AgSbO_3_ even at pressures of ≤16 GPa is likely due to the covalency of the Sb–O bonds and the moderate electronegativity of Ag^+^. The Ag–Ag distances and the channels within the Sb–O network will make this phase an ideal candidate for the exploration of ionic conductivity in the future.

## Experimental Section

### Materials

We synthesized a monoclinic AgSbO_3_ sample employing a high-pressure high-temperature (HPHT) technique with a Walker-type multianvil module. Polycrystalline ilmenite AgSbO_3_ was used as a precursor, obtained by ion exchange in solution from NaSbO_3_ and AgNO_3_ as a yellowish green color. The ilmenite AgSbO_3_ powder was placed in a Pt capsule and subjected to HPHT treatment at 5 GPa and 800 °C for 1 h, followed by quenching to room temperature and slow decompression, to obtain the red defectitve pyrochlore phase color as reported.^4^ At 16 GPa and 1380 °C, we obtained a new high-pressure phase of AgSbO_3_ (monoclinic AgSbO_3_) with a reddish-brown color at room temperature after slow decompression. The pressure and temperature were calibrated prior to the experiments by recording the resistance changes of bismuth and thermocouple calibration runs, respectively. We used the dense pellet obtained from this treatment for X-ray powder diffraction and Raman spectroscopy measurement.

### Raman Measurements

Raman scattering measurements were performed using a Horiba Jobin-Yvon LabRAM monochromator, equipped with a grating of 1800 grooves/mm and a Peltier cooled CCD camera. We used the 532 nm line of a diode laser at a power of 1 mW, and the spot diameter was ∼10 μm on the sample using a 50× microscopic objective for both focusing and collection of light. The spectral resolution was ∼1 cm^–1^ in this study. A Semrock RazorEdge filter was used to block the elastically scattered laser light, where the cutoff energy was 79 cm^–1^.

### Ellipsometry Measurements

Pressed pellets of all three phases were polished with fine sand paper (down to 2000 grit), and an ellipsometry measurement was performed on the polished surface. Room-temperature ellipsometric spectra were recorded angles of incidence of 50°, 60°, and 70° in a spectral range from 0.75 to 5 eV using a commercial ellipsometer (J. A. Woollam M-2000FI). The data collected using an angle of incidence of 70° are analyzed herein. The real and imaginary parts of the dielectric constant are converted into optical conductivity.^15^

### Band Structure Calculations

Electronic structure calculations were performed within the framework of density functional theory (DFT) as implemented in Wien2k.^16^ The generalized gradient approximation with the PBE parametrization^17^ was used. The basis set size was set to *R*_mt_*K*_max_ = 7.0, and the irreducible Brillouin zone (BZ) was sampled with a 15 × 12 × 15 k mesh. The monoclinic crystal structure (*C*2/*c* space group with *a* = 8.4570 Å, *b* = 9.8752 Å, *c* = 8.9291 Å, and β = 91.175°) was used for the high-pressure phase. The structures previously reported for the illmenite^8^ [*a* = 10.0696(1) Å, and *c* = 31.5599(1) Å] and defect pyrochlore^18^ [*a* = 19.3588(1) Å] phases were used to calculate their band structure. The muffin tin radius (*R*_MT_) for each of the elements for all three phases was set as follows: 1.25 Å for Ag, 1.04 Å for Sb, and 0.90 Å for O. The irreducible Brillouin zone (BZ) was sampled with 20 × 20 × 20 and 25 × 25 × 25 k meshes for the ilmenite phase and defect pyrochlore phase, respectively. An *R*_mt_*K*_max_ of 7.0 was used for both phases.

### Laboratory X-ray Powder Diffraction (XRPD)

XRPD measurements for structure determination under ambient conditions were performed by using a Stoe Transmission Powder Diffraction System (STADI-P) equipped with a Ge(111) Johann-type primary beam monochromator from STOE & CIE with Ag Kα1 radiation (λ = 0.55941 Å) that was equipped with an array of three linear position-sensitive MYTHEN 1K detectors from Dectris Ltd. with a 2θ opening ange of ∼18° for each. The finely powdered sample of AgSbO_3_ was placed in a 0.3 mm glass capillary (Hilgenberg glass No. 14) and spun during measurement or to improve particle statistics. The measurement in the 2θ range from 2.0° to 112° with a 2θ step width of 0.015° took 12 h (Figure 1).

For indexing of the powder pattern of monoclinic AgSbO_3_ at 298 K, TOPAS version 7 (Coelho, 2018) was used. Indexing was performed by iterative use of single-value decomposition (LSI) (Coelho, 2003), leading to a C-centered monoclinic unit cell with the following unit cell parameters: *a* = 8.4570(3) Å, *b* = 9.8752(3) Å, *c* = 8.9291(3) Å, and β = 91.1750(12)° [*V* = 745.56(4) Å^3^]. From the observed extinction rules, the most probable space group could be determined to be *Cc*, and *C*2/*c* from which the latter was confirmed after structure determination. The number of formula units per unit cell (*Z*) could be estimated to be 8 from volume increments. The peak profiles and precise lattice parameters of the powder patterns of monoclinic gSbO_3_ were first determined by a Pawley fit (Pawley, 1981) using the fundamental parameter (FP) approach of TOPAS (Cheary, Coelho, and Cline, 2005). Beforehand, the instrumental peak profile was determined using the NIST LaB6 SRM 660C line profile standard (Black et al., 2020) by applying the Thompson–Cox–Hastings pseudo-Voigt function (Thompson et al., 1987) using four line profile parameters. For the modeling of the background, Chebychev polynomials were employed. The refinement converged quickly.

The structure of high-pressure monoclinic AgSbO_3_ was determined in an iterative manner by the global optimization method of simulated annealing in real space by using TOPAS (Coelho, 2007). From the formula, space group, and number of formula units per unit cell, the following numbers of atoms in the asymmetric unit are purely arithmetically possible: two or three silver cations, two or three antimony cations, and between five and nine oxygen anions, totalling a minimum of nine and a maximum of 15 independent ions. Not all combinations make crystallographic sense. The following strategy was applied. The simulated annealing was started with a minimum of nine independent ions with the occupancy included in the simulated annealing process and a maximum number of 46 electrons, equivalent to the number for Ag^+^ or Sb^5+^. In that way, oxygen atoms could be clearly distinguished from the strong scatterers. In addition, atoms close to a special position were constrained to that position, and the occupancy factor was adjusted accordingly. A global minimum was repeatedly found with nine atoms in the asymmetric form. The distinction between the isoelectronic Ag^+^ or Sb^5+^ cations was based on their coordination spheres.

The structure giving the best fit to the data in space group *C*2/*c* was validated by Rietveld refinement (Rietveld, 1969) using TOPAS. Small amounts of AgO (∼0.9 wt %) and the AgSbO_3_ starting phase (∼0.5 wt %) were detected and included as additional phases in the Rietveld refinement. The final Rietveld refinement is shown in Figure 1. Agreement factors are listed in Table 1. The atomic coordinates are listed in Table 1, and a selection of intramolecular distances and bond angles are listed in Tables S2 and S3, respectively. The crystallographic data have been deposited at the ICSD under CSD No. 2370184 (Table 2).

**Table 2:** Comparison of Ag–O Distances in the Three AgSbO_3_ Phases

atoms	distance (Å)
*C*2/*c*	
(Ag1–O3) × 2	2.514
Ag1–O4	2.220
Ag2–O1	2.689
Ag2–O2	2.498
Ag2–O3	2.414
Ag2–O5	2.393
*Fd*3̅*mZ*	
(Ag1–O1) × 6	2.556
*R*3̅	
(Ag1–O1) × 3	2.414
(Ag1–O1) × 3	2.706

## Supporting Information Available

Atomic positions, atomic distances, bond angles, and DFT calculations for high-pressure AgSbO_3_ (PDF)
