# Supplementary material for: A New High-Pressure High-Temperature Phase of Silver Antimonate AgSbO3 with Strong Ag–O Hybridization
Source: Inorg Chem. 2024 Nov 11;63(47):22379–85. doi: 10.1021/acs.inorgchem.4c03021 (PMC11600496; doi:10.1021/acs.inorgchem.4c03021)
Supplement: Supplementary file 1 — ic4c03021_si_001.pdf [file ic4c03021_si_001.pdf]

Suppporting Information: A New High-Pressure High-Temperature Phase of Silver Antimonate $AgSbO_3$ with Strong Ag-O Hybridization

Mohamed Oudah,*,†,‡ Minu Kim,† Robert Dinnebier,† Graham McNally,† Kateryna Foyevtsova,‡ Doug Bonn,‡ and Hidenori Takagi†

†*Max Planck Institute for Solid State Research, Heisenbergstrasse 1, 70569 Stuttgart, Germany*

‡*Stewart Blusson Quantum Matter Institute, University of British Columbia, Vancouver, BC V6T 1Z4, Canada*

E-mail: mohamed.oudah@ubc.ca

Table S1: Atomic positions in the high-pressure monoclinic-$AgSbO_3$ ($C2/c$) Phase.

	x	y	z	Occ.	Site
Ag1	0.5	-0.0155(2)	0.75	1	4e
Ag2	0.8019(2)	-0.61300(16)	-0.00700(17)	1	8f
Sb1	0	0.1265(2)	0.75	1	4e
Sb2	0.6541(2)	0.68846(13)	0.12384(18)	1	8f
O1	-0.3347(13)	0.4849(12)	-0.2591(12)	1	8f
O2	0.0115(13)	0.8619(11)	-0.9691(13)	1	8f
O3	0.8440(14)	0.2681(12)	-0.2390(13)	1	8f
O4	0.5	-0.2093(17)	0.25	1	4e
O5	2.1674(14)	-1.3724(10)	-0.4809(12)	1	8f

Table S2: Select Ag-O and Sb-O distances in the high-pressure monoclinic-$AgSbO_3$ ($C2/c$) phase.

Atoms	Distance (Å)	Multiplicity
Ag1-O3	2.514	2
Ag1-O4	2.220	1
Ag2-O1	2.689	2
Ag2-O2	2.498	1
Ag2-O3	2.414	1
Ag2-O5	2.393	1
Sb1-O1	1.980	2
Sb1-O2	1.964	2
Sb1-O3	1.926	2
Sb2-O1	2.007	1
Sb2-O2	2.010	1
Sb2-O3	2.045	1
Sb2-O4	2.012	1
Sb2-O5	2.047	2

Table S3: Selected bond angles in the high-pressure monoclinic-$AgSbO_3$ ($C2/c$) phase.

Atoms	Angle (deg)
O3-Ag1-O4	148.2
O3-Ag1-O3	63.6
O3-Ag2-O5	115.2
Sb1-O1-Sb2	126.7
Sb1-O2-Sb2	130.6
Sb1-O3-Sb2	130.7
Sb2-O4-Sb2	119.8
Sb2-O5-Sb2	94.1

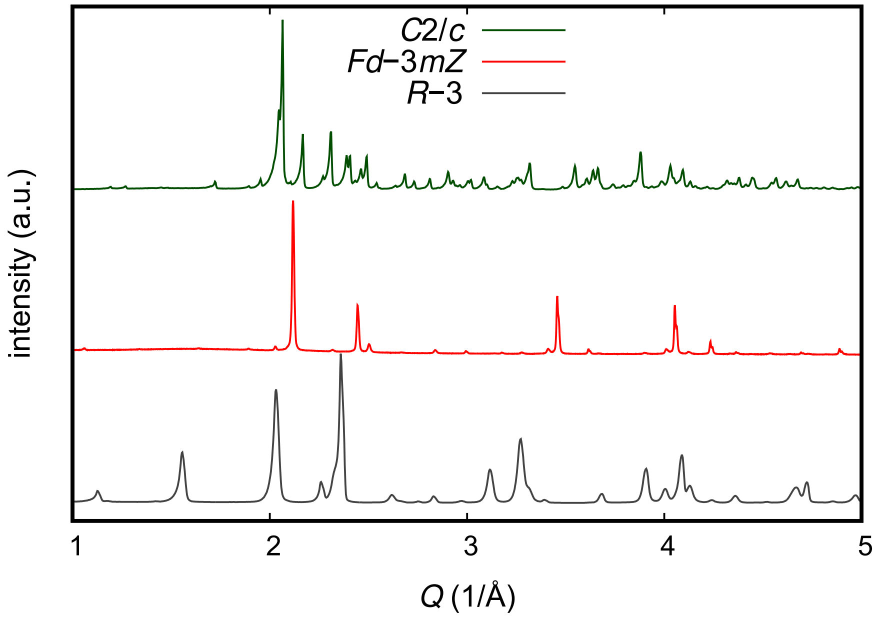

Figure S1: Comparison of x-ray diffraction pattern of the three phases of $AgSbO_3$, ilmenite (synthesized at ambient pressure, $R\overline{3}$ symmetry), defect pyrochlore (synthesized at 4 GPa, $Fd\overline{3}mZ$ symmetry), and high-pressure monoclinic-phase (synthesized at 16 GPa, $C2/c$ symmetry). All patterns were measured at ambient conditions. $R\overline{3}$ and $C2/c$ phases were measured with $Ag_{K\alpha1}$ source (0.5594 Å), while $Fd\overline{3}mZ$ phase was measured with $Cu_{K\alpha1}$ source (1.5406 Å). Data plotted as a function of Q for ease of comparison.

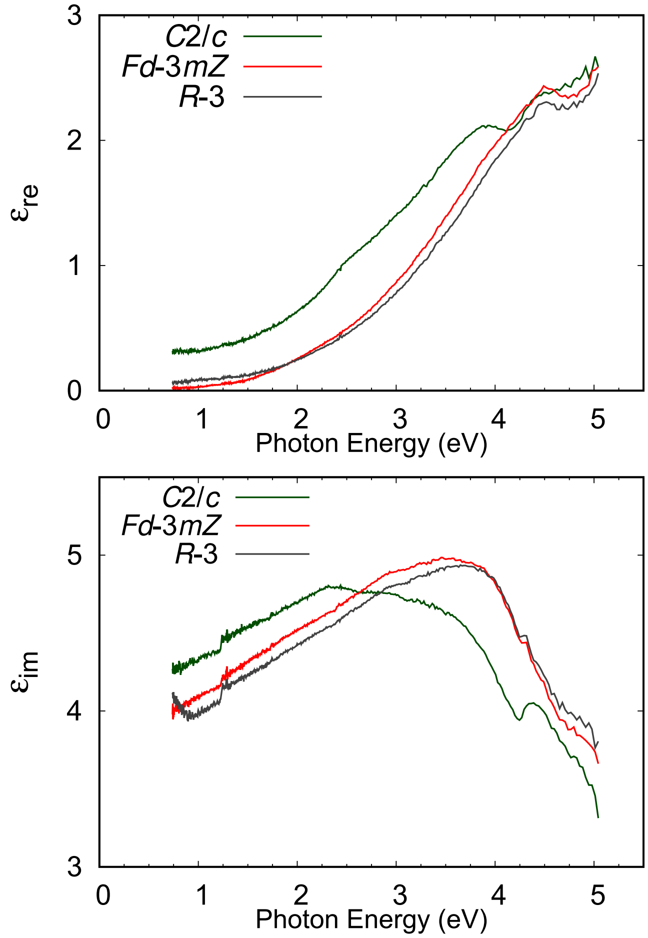

Figure S2: Real and imagniary parts of the dielectric constant of $AgSbO_3$, ilmenite (synthesized at ambient pressure, $R\bar{3}$ symmetry), defect pyrochlore (synthesized at 4 GPa, $Fd\bar{3}mZ$ symmetry), and high-pressure monoclinic-phase (synthesized at 16 GPa, $C2/c$ symmetry).

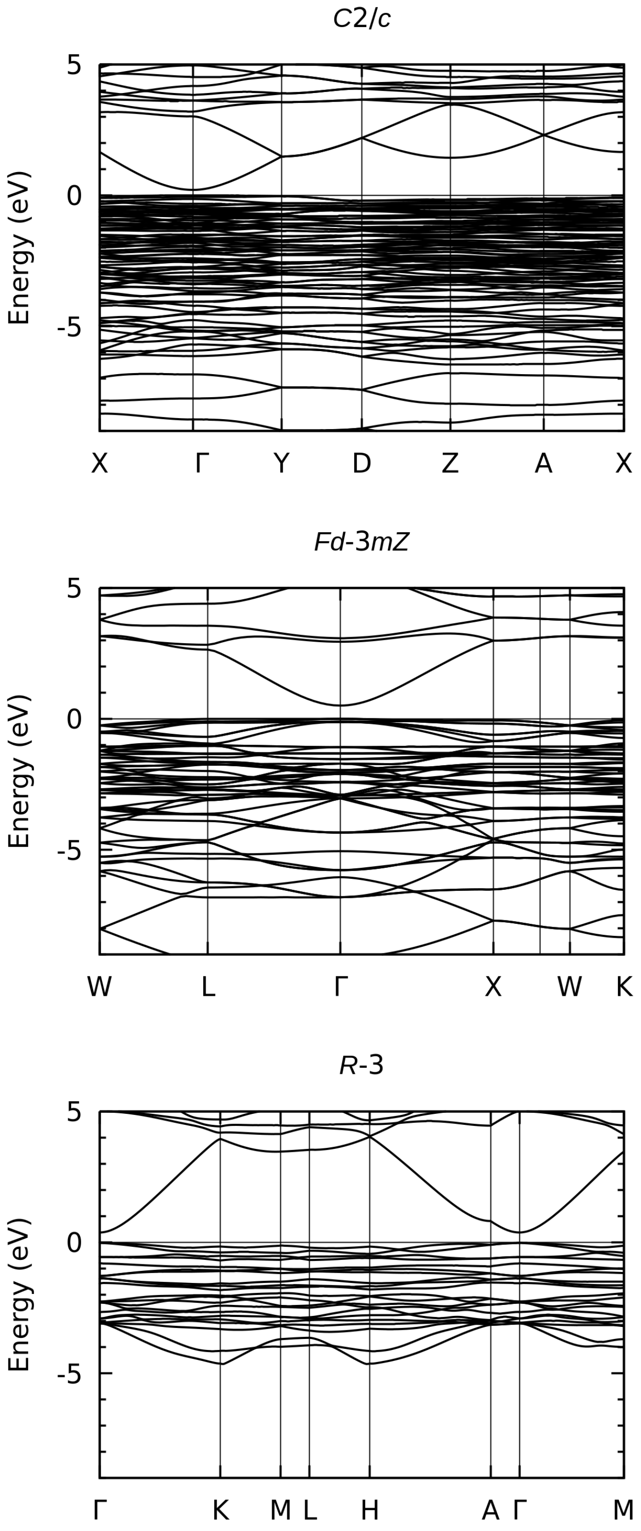

Figure S3: The calculated band structures of all three $AgSbO_3$structures, ilmenite (synthesized at ambient pressure, $R\overline{3}$ symmetry), defect pyrochlore (synthesized at 4 GPa, $Fd\overline{3}mZ$ symmetry), and high-pressure monoclinic-phase (synthesized at 16 GPa, $C2/c$ symmetry). Higher density of bands can be seen for the $C2/c$ structure reflecting the higher number atoms in the unit cell.

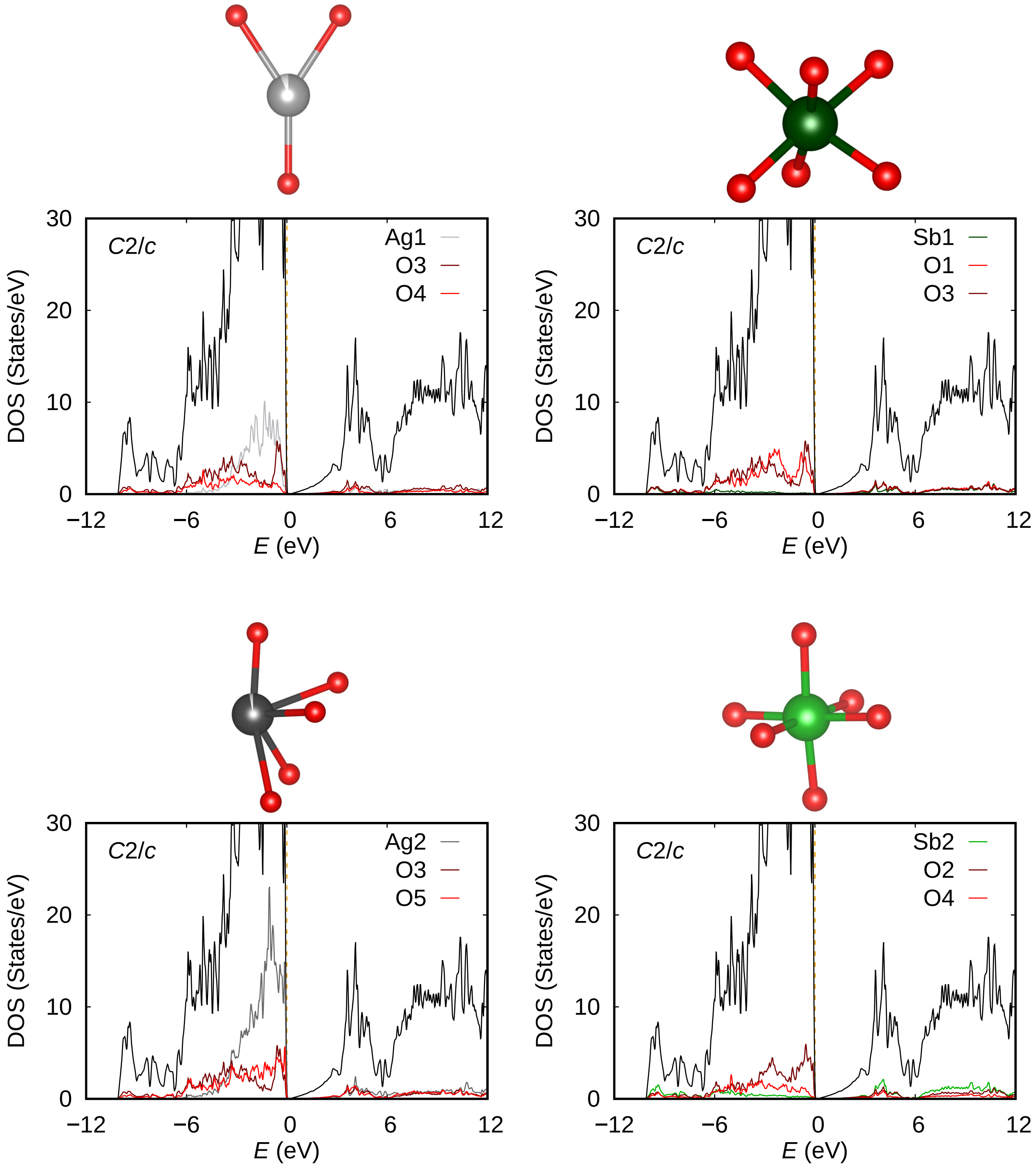

Figure S4: Partial density of states for the different unique atoms in the unit cell of the high-pressure monoclinic-phase of $AgSbO_3$ (synthesized at 16 GPa, $C2/c$ symmetry), where contribution form atoms structurally close to each other are grouped together.

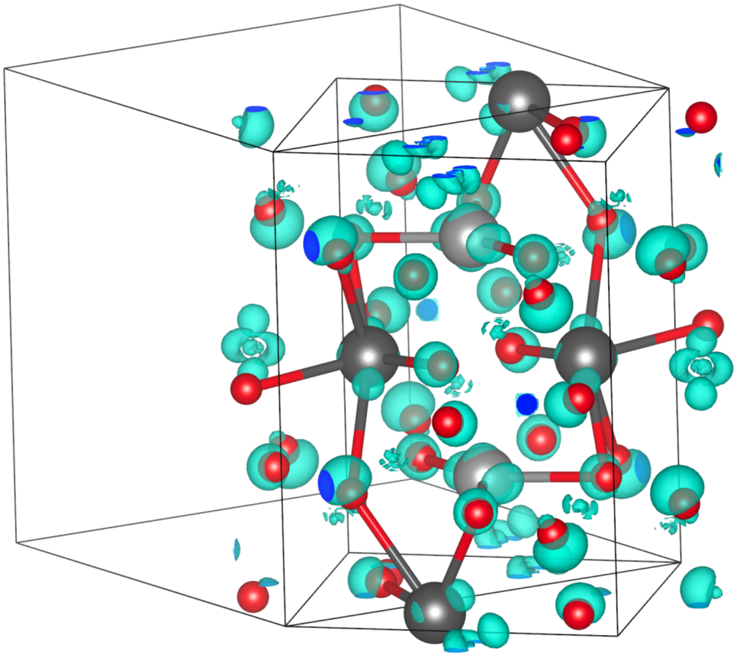

Figure S5: The absolute value of the wave function squared associated with the band above the Fermi level in the high-pressure monoclinic-phase of $AgSbO_3$ (synthesized at 16 GPa, $C2/c$ symmetry). Only the Ag and O atoms in the unit cell are shown to demonstrate that the band is dominated by MO states associated with Ag-O hybridization and O atoms part of the Sb octahedra. The two Ag1 atoms shown in light grey, while the four Ag2 atoms are shown in dark grey.
